# Trends in Route of Hysterectomy after the Implementation of a Comprehensive Robotic Training Program

**DOI:** 10.1155/2018/7362489

**Published:** 2018-09-17

**Authors:** Eleni Papalekas, Jay Fisher

**Affiliations:** Department of Obstetrics and Gynecology, Beaumont Hospital, Royal Oak, MI, USA

## Abstract

**Objective:**

To evaluate trends in surgical approach for hysterectomy following the introduction and implementation of a comprehensive robotic surgery program.

**Methods:**

A retrospective review of all hysterectomies done at two institutions, a community hospital and a suburban, tertiary-care teaching hospital, in the same health system over a five-year period, January 2010 through December 2014. A robotic surgery training program was implemented during the first year of the study and trends in route of hysterectomy were evaluated in the subsequent years.

**Results:**

A total of 5175 patients undergoing hysterectomy, for both benign and malignant indications, were included in the study. There was a significant decrease in the percent of cases performed through an abdominal approach at both the community and teaching hospitals (19.3% decline at each institution). There was an inversely related significant increase in the percent of robotic procedures at both the community and teaching hospitals (44.5% and 17%, respectively). A decrease in number of cases performed vaginally over this period was only noted in the community hospital site (25.2% decrease), and there was a slightly higher rate of vaginal hysterectomies at the teaching hospital over this study period (21.9% in 2010, 24.1% in 2014).

**Conclusion:**

The decrease in number of abdominal and laparoscopic hysterectomies and increase in number of robotic hysterectomies that was seen are consistent with national trends. The initiation of a robotic training program did not prevent the proliferation of use of the robot but did aim to ensure proficiency on the robot prior to gaining privileges for patient use. This type of comprehensive training and monitoring program could be applied to future technologic advances to ensure a standard level of surgical proficiency. Trends in route of hysterectomy are clearly multifactorial and involve patient, provider, and location-specific factors that are likely to continue to change.

## 1. Introduction

Hysterectomies are the most common gynecologic surgical procedures in the United States with an estimated number of 600,000 cases performed annually [1, 2]. While the procedure has traditionally been performed either by laparotomy or straight-stick (also known as “traditional”) laparoscopy or vaginally, the introduction of the robotic-assisted hysterectomy offers patients another minimally invasive option. With the US Food and Drug Administration approval of a robotic mode of hysterectomy in 2005, the da Vinci surgical platform was introduced [3], offering women smaller incisions, shorter hospital stays, and the potential for fewer complications when compared to abdominal hysterectomy. However, the translation of this technology into practice leaves providers responsible for determining the optimal patients for each surgical approach. According to ACOG, a vaginal hysterectomy should be performed “whenever feasible” [1, 4]. When it is not feasible, surgeons are left to choose a different surgical approach.

Prior to the introduction of the robotic mode of hysterectomy, if the vaginal approach was not possible, a traditional laparoscopic approach was often considered. However, traditional laparoscopy requires the development of very specific surgical skills associated with a steep learning curve. The main complaints about traditional laparoscopy include counter-intuitive hand movements, 2D visualization, and limited degrees of instrument motion within the body [5].

Robotic gynecologic surgery has generated a great deal of excitement: offering more “wrist-like” motions and 3D visualization and enabling the surgeon to be seated at a console remote from the patient. However, existing data on outcomes of robotic-assisted hysterectomies is limited. A 2014 Cochrane review of robotic surgery for gynecologic disease found six RCTs with 371 women undergoing hysterectomy via either robotic-assisted or conventional laparoscopic techniques. According to this study, there were no measurable differences in either of the primary outcomes, which included intraoperative complications and postoperative complications [4, 6, 7]. Based on these systematic reviews and other available data, there is not enough evidence to make definitive conclusions regarding robotic-assisted compared to conventional laparoscopic hysterectomy [3–5, 8, 9]. Nonetheless, gynecologists have incorporated this technology into their armamentarium at a much greater rate than traditional laparoscopic procedures which were adopted, and they are left to choose the route of hysterectomy using current research, personal surgical expertise, and individual patient characteristics to guide their decision.

## 2. Materials and Methods

This was a retrospective repeated cross-sectional study. In this multisite study, information was collected from two separate hospitals within the Beaumont Health System. The first site is Beaumont Royal Oak, a 1,070-bed tertiary-care center that performs approximately 48,000 surgeries per year. It is considered a teaching hospital with 24 Ob/Gyn residents and full subspecialty services. The second site is Beaumont Troy, a 458-bed private community hospital without residents and with limited subspecialty consulting services. The two hospitals are very similar in terms of demographic and socioeconomic patient populations. Subjects undergoing hysterectomy between January 1, 2010, and December 31, 2014, were included in the study. 2010 was chosen as the first year of the study because this was when the robotic surgery training program and eligibility guidelines were initially implemented. A study period of five years was then chosen to adequately track the effects of these changes, represented by the rates in route of hysterectomy, comparing the first year, prior to full implementation, to the subsequent years.

Subjects to be included in the study were identified by Current Procedural Terminology (CPT) codes. All codes containing the word “hysterectomy” were used to generate the list of patients. Patients who underwent hysterectomy for any indication were included.

Hysterectomy is defined as the surgical removal of the uterus. In this study the types of hysterectomy were categorized as follows:

- Abdominal hysterectomy (AH) is accomplished by creating an incision in the abdomen through which the entire surgery is carried out and uterus removed.
- Vaginal hysterectomy denotes a procedure mainly performed transvaginally, including the removal of the uterus. The two subtypes are as follows:
  - Vaginal hysterectomy (VH), where the entire procedure is performed transvaginally without the assistance of any abdominal incisions.
  - Laparoscopic-assisted vaginal hysterectomy (LAVH), where the division of the uterine vessels is performed transvaginally but laparoscopy assists in the procedure to some extent. In the vast majority of cases, laparoscopy was utilized to perform unilateral or bilateral salpingooophorectomy. Thus the surgical skills needed to perform a vaginal hysterectomy were still employed, making it most appropriate to categorize them amongst the other vaginal hysterectomies.
- Laparoscopic hysterectomy refers to a procedure that is largely carried out via conventional (straight-stick) laparoscopy. The subtypes are as follows:
  - Laparoscopic supracervical hysterectomy in which the uterine vessels are ligated laparoscopically and the cervix is amputated at the level of the uterine vessels.
  - Total laparoscopic hysterectomy (TLH) where the entire procedure, including the suturing of the vaginal cuff, is carried out with conventional laparoscopy.
- Robotic-assisted hysterectomy, both subtypes below are utilizing the da Vinci surgical platform as the mode of laparoscopy. Subtypes include the following:
  - Robotic-assisted total laparoscopic hysterectomy (RATLH), which consists of removal of cervix, uterus, and bilateral adnexal structures using the robot.
  - Robotic-assisted supracervical hysterectomy (RASCH), which consists of removal of uterus and adnexal structures above the level of the cervix

A comprehensive robotic surgery training and monitoring program was initiated during the first year of the study. Because full implementation did not occur until the end of the first year, 2010 served as an index year to compare to subsequent years during which the training program was fully functional. The program entailed robotic simulation, mentoring, proctoring, and maintenance of skills (Box 1). More specifically, surgeons with a projected volume of > 15 robotic or advanced laparoscopic (which could include hysterectomy, myomectomy, sacrocolpopexy, and extensive excision of endometriosis) cases per year were eligible for the program. The first step in training was to complete online learning modules. These modules are found on the da Vinci website and are designed to teach about the robot and its components, setting up the equipment and using/understanding basic principles of electrosurgery. Once completed, trainees had to complete simulation modules on the da Vinci or Mimic consoles. Each module had to be completed with a passing rate of 70% or higher. Next, trainees had to observe at least two robotic-assisted hysterectomies. The proctor and training physician then performed three to five cases jointly with shared time on the robotic console. The last step in the training program was three or more monitored cases. Training physicians were expected to complete all steps of training within a six-month time period. After successful completion, surgeons were granted probationary privileges for one year. After the first year, a QA committee reviewed the surgeon's case volume and patient outcomes and subsequently granted or denied continued privileges.

## 3. Results

Patients who had a hysterectomy for any indication were identified and included in the study. Combining data from both hospitals, there were 5175 cases total: 1036 cases in 2010, 1072 cases in 2011, 1054 cases in 2012, 999 cases in 2013, and 1014 cases in 2014. These patients were stratified into a category based on type of hysterectomy performed and percentages were calculated and trended for further analysis.

**Figure 1:**
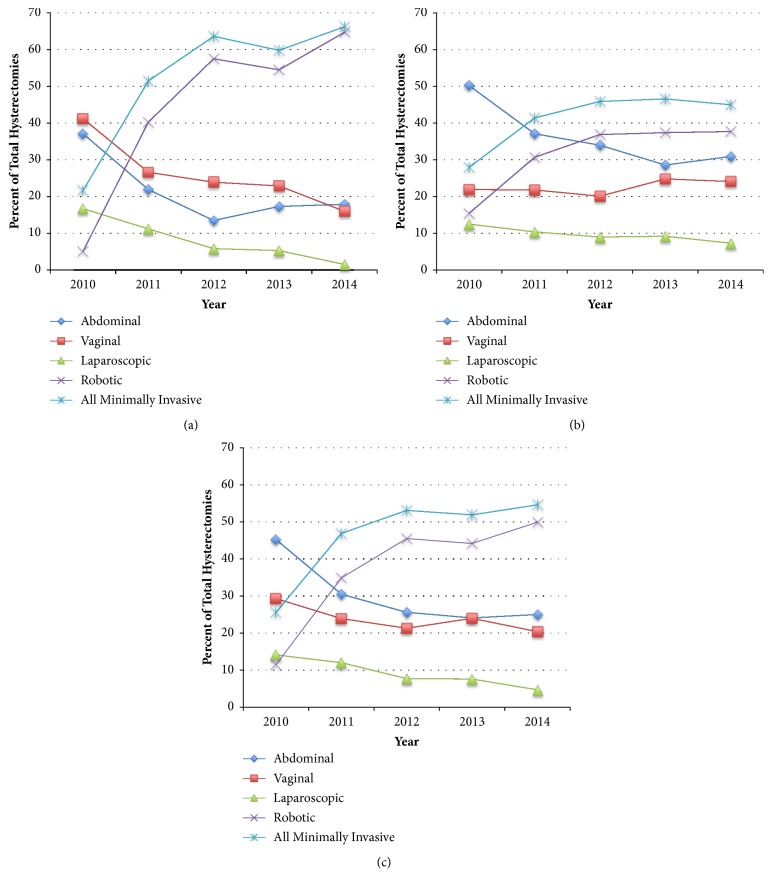
(a) Percent of hysterectomies by route-community hospital. (b) Percent of hysterectomies by route-teaching hospital. (c) Percent of hysterectomies by route-both hospitals.

Over the five-year study period there was a significant decrease in the percent of cases performed via an abdominal approach. In the community hospital the percent of abdominal hysterectomies went from 37.1% in 2010 to 17.9% in 2014 (Table 1, Figure 1(a)). In the teaching hospital the rate of abdominal hysterectomies went from 50.2% in 2010 to 30.9% in 2014 (Table 1, Figure 1(b)). Looking at the hospitals together, there was 20.2% overall decline in the rate of abdominal hysterectomies over the study period (Table 2, Figure 1(c)).

The rate of nonrobotic laparoscopic cases also decreased. In the community and academic hospitals only 16.7% and 12.5% (respectively) of hysterectomies were done via this method at the beginning of the study period. Despite already being the least commonly used method of hysterectomy, rates of “straight-stick” laparoscopy were seen to further decline. In the community hospital, the rate fell by 15.2%, with this route making up less than 2% of hysterectomies in 2014 (Table 1, Figure 1(a)). In the academic hospital the rate of this approach decreased by 5.2% (Table 1, Figure 1(b)). In both hospitals together, there was a 9.4% decline (Table 2, Figure 1(c)).

There was a concurrent, inversely related, significant increase in the rate of robotic cases. This was seen in both the community hospital, where robotic cases rose by 59.6% (Table 1, Figure 1(a)), and the teaching hospital, where robotic cases rose by 22.2% (Table 1, Figure 1(b)). Combining the data from both hospitals, the rate of robotic cases rose by 38.4% (Table 2, Figure 1(c)).

A decrease in the number of cases done vaginally was noted at the community hospital only, where there was a 25.2% decline during the study period (Table 1, Figure 1(a)). In the teaching hospital, the percent of cases performed vaginally was 21.9% in 2010 and increased (although not achieving statistical significance) to 24.1% of cases in 2014 (Table 1, Figure 1(b)).

## 4. Discussion

The introduction of robotic surgery has had a significant impact on trends in route of hysterectomy. In this hospital system, the implementation of a comprehensive robotic training and monitoring program aimed to ensure proficiency prior to its use. There was a significant decline in percent of abdominal hysterectomies performed, with an inversely related increase in laparoscopic hysterectomies, specifically robotic-assisted, over the same time period. These results are consistent with a study published in JAMA in 2013 that gathered data from 441 hospitals across the US (N = 264,758) [3]. This study had a four-year study period, from 2007-2010, and found that there was a substantial increase in both laparoscopic and robotic-assisted hysterectomies, and a decrease in both vaginal and abdominal hysterectomies. While the results of our study are similar to previously published data, it also gives us insight as to how different hospital settings may impact these trends [3, 10–13]. One unique strength of our study is the inclusion of data from a large teaching hospital in addition to a smaller community hospital. As a result, our data can be extrapolated to larger patient populations. This also helps to ensure that observed trends are not unique to an academic center that may reflect a different rate of adoption than that of community hospitals. In addition, this study also provides a relatively long study period of five years over which to monitor these trends.

The ability to analyze data from the two hospitals separately has its own advantages. In the community hospital the rates of vaginal hysterectomy decreased over the study period, whereas in the academic hospital the rates increased. One plausible explanation is that practice is more influenced by consumer demand in the private hospital setting. Advertisements and publicity permeate the minds of the general public, seemingly insinuating that robotic surgery is the current standard of practice. Vaginal hysterectomies are simultaneously viewed as less desirable and outdated. On the contrary, an academic center has a strong emphasis on education and evidence-based practice standards. In this setting, surgical technique is taught and generally performed in accordance with current practice guidelines. The rate of vaginal hysterectomies remained unchanged over the study period in keeping with ACOG recommendations to do so whenever feasible. In addition, full subspecialty services at the academic center, including urogynecology, are available to assist and proctor surgeons performing vaginal cases. All of these factors help explain the disparity in rates of vaginal hysterectomies in the different hospital settings.

A weakness of the study is that the design type is observational in nature and does not provide causality for the observed trends. Deciding upon a route of hysterectomy involves both surgeon and patient-specific factors. The main surgeon-specific factor addressed in this study is the surgeon's skill set and comfort level with traditional laparoscopic and/or robotic surgery, compared to conventional vaginal approach. The robotic training and certification program in this study allowed surgeons to learn and subsequently elect to perform robotic hysterectomy if they were interested in doing so. Conversely, practitioners who do not routinely perform vaginal hysterectomies may not be comfortable employing this approach even when surgically indicated. Other surgeon-specific factors, such as access to laparoscopic equipment and compensation for different routes of hysterectomy, add an additional layer of complexity in surgical planning. Patient-specific factors include pathology and patient-specific characteristics (i.e., obesity) and how they have changed over time. Finally, cost is a driving force in all aspects of healthcare and greatly impact trends in surgical methods. Some insurance companies require preauthorization for any nonvaginal hysterectomy [14]. While this study investigated overall trends in route of hysterectomy, the next step may be to further analyze causation and stratify the impact of these recognized factors.

An interesting implication of the changing rates in route of hysterectomy is how this will affect future Ob/Gyn resident training. From 2003 to 2011, the American Council of Graduate Medical Education (ACGME) reported a decline in the mean number of abdominal and vaginal hysterectomies per resident, by 27.4% and 43%, respectively [15, 16]. This trend is consistent with the data from the community hospital, where a decline in both vaginal and abdominal hysterectomies was observed. This translates into fewer open abdominal and vaginal hysterectomies, which subsequently threatens the development of broad-based surgical skills in training gynecologists. However, we found no change in the rate of vaginal hysterectomies performed at the academic center during the study period. Despite the national trend, it is clearly possible to maintain numbers of vaginal cases alongside the increasing popularity of the robot. It is the responsibility of practicing gynecologists to acknowledge vaginal hysterectomy as the ACOG-endorsed standard of care so it does not get underutilized or forgotten. While not feasible in every patient, this route should be discussed and offered when possible. If current trends continue undeterred, development of vaginal and abdominal surgical skills in training gynecologists will be significantly affected.

## 5. Conclusion

Whether viewed from a patient, resident, or attending perspective, the introduction of the robotic-assisted hysterectomy has significantly impacted the field of gynecology. How this skill set will ultimately fit into the toolbox of the practicing gynecologist is yet to be determined. The robotic training and monitoring program detailed in this study offers an effective way to ensure surgical proficiency of providers offering robotic services. Perhaps more significantly, in a teaching hospital setting, it is more feasible to address the appropriate application of new technologies while offering support for less experienced surgeons. This would include subspecialty support, such as urogynecology, for potential vaginal hysterectomy candidates. Trends in route of hysterectomy are clearly multifactorial and involve patient, provider, and location-specific factors that are likely to continue to change.

## Figures and Tables

**Box 1:**
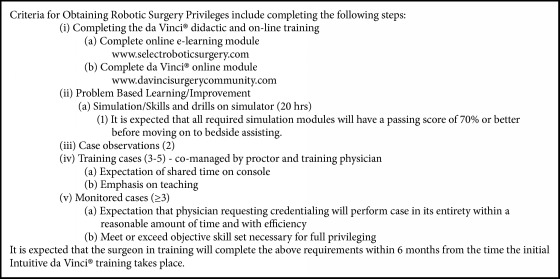
Robotics curriculum.

**Table 1:** Percent of hysterectomies by route-by hospital.

	**Hospital Type**	**2010 (n)**	**2011 (n)**	**2012 (n)**	**2013 (n)**	**2014 (n)**	%** Change 2010- 2014**
**Abdominal**	Community	37.1 (147)	21.9 (102)	13.5 (58)	17.3 (69)	17.9 (84)	-19.3 (*∗*)
	Teaching	50.2 (321)	37.1 (225)	34 (212)	28.6 (172)	30.9 (169)	-19.3 (*∗*)

**V** **a** **g** **i** **n** **a** **l** ^**a**^	Community	41.2 (163)	26.6 (124)	23.9 (99)	22.9 (91)	16.0 (75)	-25.2 (*∗*)
	Teaching	21.9 (140)	21.8 (132)	20.1 (125)	24.8 (149)	24.1 (132)	+2.3

**L** **a** **p** **a** **r** **o** **s** **c** **o** **p** **i** **c** ^**b**^	Community	16.7 (66)	11.2 (52)	5.8 (25)	5.3 (21)	1.5 (7)	-15.2 (*∗*)
	Teaching	12.5 (80)	10.4 (63)	9 (56)	9.2 (55)	7.3 (40)	-5.2 (*∗*)

**Robotic**	Community	5.1 (20)	40.3 (188)	57.5 (249)	54.5 (217)	64.7 (304)	+59.6 (*∗*)
	Teaching	15.5 (99)	30.7 (186)	36.9 (230)	37.4 (225)	37.7 (206)	+22.2 (*∗*)

**All Minimally ** **I** **n** **v** **a** **s** **i** **v** **e** ^**c**^	Community	21.7 (86)	51.5 (240)	63.6 (274)	59.8 (238)	66.2 (311)	+44.5 (*∗*)
	Teaching	28.0 (179)	41.4 (249)	45.9 (286)	46.6 (280)	45.0 (246)	+17.0 (*∗*)

Percentages are rounded up to the nearest tenth place; a includes laparoscopic-assisted vaginal hysterectomy; b includes total laparoscopic hysterectomy and laparoscopic supracervical hysterectomy both via traditional laparoscopy; c combined robotic and laparoscopic cases; *∗*statistical significance, p < 0.05.

**Table 2:** Percent of hysterectomies by route-both hospitals.

**Type of Hysterectomy**	**2010 (n)**	**2011 (n)**	**2012 (n)**	**2013 (n)**	**2014 (n)**	%** Change 2010-2014**
**Abdominal**	45.2 (468)	30.5 (327)	25.6 (270)	24.1 (241)	25.0 (253)	-20.2 (*∗*)

**V** **a** **g** **i** **n** **a** **l** ^**a**^	29.3 (303)	23.9 (256)	21.3 (224)	24.0 (240)	20.4 (207)	-8.8 (*∗*)

**L** **a** **p** **a** **r** **o** **s** **c** **o** **p** **i** **c** ^**b**^	14.1 (146)	12 (115)	7.7 (81)	7.6 (76)	4.7 (47)	-9.4 (*∗*)

**Robotic**	11.5 (119)	34.9 (374)	45.5 (479)	44.2 (442)	49.9 (506)	+38.4 (*∗*)

**All Minimally ** **I** **n** **v** **a** **s** **i** **v** **e** ^**c**^	25.6 (265)	46.9 (489)	53.1 (560)	51.9 (518)	54.6 (554)	+29.1 (*∗*)

Percentages are rounded up to the nearest tenth place; a includes laparoscopic-assisted vaginal hysterectomy; b includes total laparoscopic hysterectomy and laparoscopic supracervical hysterectomy both via traditional laparoscopy; c combined robotic and laparoscopic cases; *∗*statistical significance, p < 0.05.

## Data Availability

The data used to support the findings of this study are included within the article.
